# Regulation of α5 and αV Integrin Expression by GDF-5 and BMP-7 in Chondrocyte Differentiation and Osteoarthritis

**DOI:** 10.1371/journal.pone.0127166

**Published:** 2015-05-26

**Authors:** David Garciadiego-Cázares, Hilda I. Aguirre-Sánchez, René F. Abarca-Buis, Juan B. Kouri, Cristina Velasquillo, Clemente Ibarra

**Affiliations:** 1 Unidad de Ingeniería de Tejidos, Terapia Celular y Medicina Regenerativa, Instituto Nacional de Rehabilitación (INR), Secretaría de Salud (SSA), Mexico City, Mexico; 2 Laboratorio de Tejido Conjuntivo, Instituto Nacional de Rehabilitación (INR), Secretaría de Salud (SSA), Mexico City, Mexico; 3 Departamento de Infectómica y Patogénesis Molecular, Centro de Investigación y de Estudios Avanzados, Instituto Politécnico Nacional (CINVESTAV-IPN), Mexico City, Mexico; University of Massachusetts Medical, UNITED STATES

## Abstract

The Integrin β1 family is the major receptors of the Extracellular matrix (ECM), and the synthesis and degradation balance of ECM is seriously disrupted during Osteoarthritis (OA). In this scenario, integrins modify their pattern expression and regulate chondrocyte differen-tiation in the articular cartilage. Members of the Transforming growth factor beta (Tgf-β) Su-perfamily, such as Growth differentiation factor 5 (Gdf-5) and Bone morphogenetic protein 7 (Bmp-7), play a key role in joint formation and could regulate the integrin expression during chondrocyte differentiation and osteoarthritis progression in an experimental OA rat model. Decrease of α5 integrin expression in articular cartilage was related with chondrocyte dedif-ferentiation during OA progression, while increase of α1, α2, and α3 integrin expression was related with fibrous areas in articular cartilage during OA. Hypertrophic chondrocytes expressedαV integrin and was increased in the articular cartilage of rats with OA. Integrin expression during chondrocyte differentiation was also analyzed in a micromass culture system of mouse embryo mesenchymal cells, micromass cultures was treated with Gdf-5 or Bmp-7 for 4 and 6 days, respectively. Gdf-5 induced the expression of theα5 sub-unit, while Bmp-7 induced the expression of the αV sub-unit. This suggests a switch in signaling for prehypertrophic chondrocyte differentiation towards hypertrophy, where Gdf-5 could maintain the articular chondrocyte phenotype and Bmp-7 would induce hypertrophy. Decrease of *Ihh* expression during late stages of OA in rat model suggest that the ossification in OA rat knees and endochondral ossification could be activated by Bmp-7 and αV integrin in absence of Ihh. Thus, chondrocyte phenotype in articular cartilage is similar to prehypetrophic chondrocyte in growth plate, and is preserved due to the presence of Indian hedgehog (Ihh), Gdf-5 and α5 integrin to maintain articular cartilage and prevent hy-pertrophy.

## Introduction

Articular cartilage is a poorly regenerative tissue, and an injury in articular cartilage generally results in Osteoarthritis (OA) with the following three relevant characteristics: loss of synovial space; cartilage vascularization, and osteophyte formation [1,2]. It was previously thought that osteophytes were formed mainly at the edge of articular cartilage with the initiation of bone invasion into the cartilage. However, several studies have shown that osteophytes could be the result of differentiation toward hypertrophy (endochondral ossification) [3], as occurs in the growth plate during skeletogenesis. Chondrocytes are the sole cell type in articular cartilage and in growth plate cartilage, but they are quite different [4]. The proliferation rate is lower in articular chondrocytes, and this characteristic prevents them from becoming hypertrophic chondrocytes and inhibits articular cartilage ossification during their life span.

β1-integrin family adhesion molecules comprise the main Extracellular matrix (ECM) receptors; two transmembrane sub-units (alpha and beta) form a functional dimer. Integrins regulate the proliferation, migration, survival, and differentiation of several cell types. It has been recognized that the ligand for integrins α1-, α2-, α3-, and α10β1 is collagen, while for integrin α5β1 the ligand is fibronectin, and for α6β1, this is laminin [5]. All of these integrins are expressed dynamically during chondrocyte differentiation [6–11]. β1-integrin knockout mice result in very early embryonic lethality, and β1-integrin-deficient chondrocytes had an abnormal shape, failed to arrange into columns in the growth plate, and exhibited decreased proliferation due to a defect in G1/S transition and cytokinesis [12]. In addition, blocking the function of β1, α2, and α3 integrin sub-units inhibits hypertrophic differentiation, and chondrocyte survival decreases [13]. The α9β1 integrin regulates osteoclast formation [14], and the αVβ1 integrin promotes endochondral ossification-recruiting blood vessels [15], which carry the osteoblast for bone formation. The α5β1 integrin plays a particular role during the establishment of skeletal elements, blocking α5β1 integrin activity during skeletal development and resulting in ectopic joint formation, demonstrating that α5β1 integrins coordinate joint formation and cartilage differentiation in appendicular skeleton [16]. The appendicular skeleton is formed initially by cartilage; *Sox-9* expression is a cell marker for mesenchymal commitment toward a chondrogenic lineage [17]. During skeleton morphogenesis, cartilage anlage, composed mainly of proliferating chondrocytes, becomes apparent in early limb budding, forming uninterrupted Y-shaped mesenchymal condensation, branches and finally, the formation of a joint [18]. The first sign of joint development is the expression of *gdf-5* and *wnt-9a* limited to the interzone [19,20], the region where joints will be formed. The interzone consists of compact and closely packed mesenchymal cells. If any of these molecules are suppressed, joints are not formed. The α5β1 integrin is also found in cartilage anlage, which is composed mainly of proliferating chondrocytes, but its expression is considerably inhibited at the interzone [16]. The presence of the α5β1 integrin in perichondrium and cartilage anlage promotes cell proliferation and chondrocyte differentiation to a prehypertrophic fate through members of the Bmp family, which induce the expression of Indian hedgehog (Ihh), a factor that promotes cell proliferation and chondrocyte prehypertrophy [21].

It is known that both the α5β1 integrin and the *Ihh* gene are co-expressed in prehypertrophic chondrocytes, allowing their differentiation toward hypertrophy and leading to endochondral ossification. This is not the case at the interzone, where cells loose the chondrocyte-type phenotype and this cell population could be the source of all of the joint cell types, including articular chondrocytes. Endochondral ossification occurs mainly at the longitudinal growth plate of long bones. The BMP family is involved in different steps of chondrogenesis as follows: in the compaction of mesenchymal cell aggregations that outline the skeletal elements; in cartilage differentiation from the prechondrogenic mesoderm, and in the establishment of prehypertrophic chondrocytes [21,22]. In this late stage of chondrocyte differentiation, BMP that are expressed in the perichondrium induce Parathyroid hormone related protein (PTHrP) expression and Ihh to maintain the prehypertrophic state of chondrocytes [23,24]. A good candidate for the response of this signaling is Bmp-7, due to perichondrium expression during chick appendicular skeleton development [25]. GDF-5 is another member of the BMP family that is expressed in developing joints and that plays a key role in joint specification of synovial joints [26], because *gdf-5* knockout mutation in mice and its homolog in humans, cartilage-derived morphogenetic protein-1 (CDMP-1) showed alterations in joint morphogenesis [19]. The absence of α5β1 integrin in the interzone permits progression of joint development, and Bmp signaling regulates the cartilage differentiation during joint development. Thus, the α5β1 integrin could controls the cell response mediated by Bmp to prehypertrophic fate or joint fate blocking expression of *Wnt-9a*, considered a joint inducer and inducing *Ihh* expression. Despite the role of α5β1 integrin in joint morphogenesis and specification, there are few studies on the role of the β1-integrin family in the development and maintenance of articular cartilage. Bmp-7 is a strong inducer of endochondral ossification and is expressed in the perichondrium/periosteum during skeletogenesis, but it is not expressed in the interzone [25]. We suggest that Gdf-5 and Bmp-7 might contribute to knowledge of the functional differences between articular cartilage and growth plate cartilage, because the former is not influenced by Bmp-7 signals, as compared with growth plate cartilage. In contrast, like in the interzone the articular cartilage is under the influence of the Gdf-5 signal.

One of the many diseases that cause degeneration of articular cartilage in adults is OA, the role of integrins during the progress of OA has been reported. The α1 integrin knockout exhibits OA with increased levels of Matrix metalloproteinase (MMP)-2 and -3, low levels of proteoglycans, synovial hyperplasia, and reduced cartilage cellularity [27]. Adult human articular chondrocytes express different heterodimers of the β1-integrin family, with prominent expression of α5β1 [28] accompanied by α1β1 [29], αVβ1, and lower levels of α3β1 and αVβ3 [30]. All of these integrins increase their expression in human and bovine chondrocytes from osteoarthritic cartilage.

Previous works showed that integrins regulate chondrocyte differentiation and joint formation by blocking the expression of *Wnt-9a* and inducing the expression of *Ihh*; additionally, the expression of integrins in the articular cartilage might be regulated by growth factors, such as Transforming growth factor beta (Tgf-β), Growth differentiation factor 5 (Gdf-5), and Bone morphogenetic protein 7 (Bmp-7), and by Extracellular matrix (ECM) components. Because the integrins controls chondrocyte maturation, we found that α5 integrin expression decreases and αV integrin expression increases in the early stages of OA. We also find that *Ihh* increase its expression in the earlier stages of OA, and at the late stages of OA decrease its expression in a similar way toα5 integrin expression, and articular cartilage rapidly differentiates into bone in a process like endochondral ossification in OA.

## Materials and Methods

### Reagents

The following antibodies were used: anti-β1 integrin (L-16); anti-α1 integrin (R-19); anti-α2 integrin (N-19); anti-α3 integrin (C-18); anti-α5 integrin (P-19); anti-αV integrin (N-19), and anti-Gdf5 (N-17) polyclonal antibodies for immunohistochemistry and immunofluorescence, which were purchased from Santa Cruz Biotechnology (Santa Cruz, CA, USA). Anti-type I collagen (690F) and type II collagen (690F) were purchased from ICN Biomedical, Inc. (Aurora, OH, USA), anti-aggrecan (MCA1451) was from AbD SeroTec (Raleigh, NC, USA), and anti-fibronectin (F14420) was purchased from Transduction Laboratory. BMP-7 (120–03) and GDF-5 (120–01) were purchased from PeproTech (Rocky Hill, NJ, USA). Dulbecco’s modified Eagle Medium (DMEM) and BGJb culture media were obtained from Invitrogene (Grand Island, NY, USA).

### Osteoarthritis Experimental Model

All animals were treated in accordance with the Public Health Service Policy on Humane Care and Use of Laboratory Animals (August 2002), implemented by the Office of Laboratory Animal Welfare, Harvard Medical School (American Association for Laboratory Animal Science [IACUS]). Rats were kindly donated by the animal facility of CINVESTAV-IPN (Mexico City, Mexico). Carbon dioxide (CO_2_) euthanasia was performed in rats. The Mexican Internal Committee for the Care and Use of Laboratory Animals (Comité Interno para el Cuidado y Uso de Animales de Laboratorio, CICUAL) of the Instituto Nacional de Rehabilitación (INR) in Mexico City approved this study (Approval number 05/09).

For the experimental model, 30 Wistar male rats 20 days of age were obtained from the animal facility of CINVESTAV-IPN. In order to induce experimental OA, a partial meniscectomy was performed on the right knees of 15 Wistar male rats, as described elsewhere [31]. The rats were progressively exercised to accelerate articular damage for 5, 10, and 20 days. The remaining 15 rats without surgery and without exercise were employed as normal controls. After 5, 10, and 20 days, rats from both groups were sacrificed (carbon dioxide (CO_2_) euthanasia was performed) prior to knee dissection; knees dissected were fixed in 4% PFA overnight. Then, the tissues were dehydrated and paraffin-embedded for preparing histological sections.

### Histology and Tissue Staining

Five-μm-thick sections were stained with Safranin O and Fast Green for cartilage tissue analysis and with Herovici stain for collagen replacement in tissues during OA. Herovici staining was performed for the qualitative degree of collagen cross-linking in order to discriminate between young and mature collagen, and a mixture of picric acid, methyl blue, and acid fuchsine was used to distinguish type I from type III collagen. The remaining tissue sections were utilized for immunohistochemistry, immunofluorescence, and *in situ* hybridization.

### Immunohistochemistry

For immunohistochemical analysis, slides and micromass cultures were incubated overnight with antibodies at 4°C. The following morning, slides were incubated with the corresponding Fluorescein (FITC)-labeled secondary antibody or biotin conjugate for 2 h at 37°C, observed under a Zeiss Fluorescence Axio Imager A-1 Microscope, and photographed with an Axio Vision Hrc Digital Camera (Zeiss, Germany).

### Chondrogenesis Model

The micromass cultures were obtained from CD1 mouse embryos 11.5 days postcoitum (E11.5) from the Animal Care Center of Instituto de Investigaciones Biomédicas (UNAM), and the Animal Care Center of Instituto Nacional de Rehabilitación. Cells from limb-bud embryos were digested with 0.3% trypsin for 15 min and then with 0.3% collagenase for 20 min at 37°C. Mesenchymal cells were seeded at a density of 20,000 cells in 10 μL of culture medium in 24-well plates and incubated for 1 h. Then, 500 μL of culture medium was added. After 3 days, the cells formed cartilaginous nodules and the effect of growth factors on micromasses was tested. This medium was changed for fresh medium containing 100 nM Gdf-5 or Bmp-7. Three days later, the culture was analyzed by immunohistochemistry to determine the expression of different integrin subunits and ECM-type molecules of articular cartilage.

### Statistical Analysis

For micromass, Alcian blue staining, and immunohistochemistry analysis, the photographs of micromass cultures for each treatment (control, (Transforming growth factor beta [TGF-β]), Bone morphogenetic protein 7 [BMP-7], and Growth differentiation factor [GDF-5]) were analyzed. Analysis of the percentage of stained area of the total area (500 μm^2^) was performed using ImageJ software, and the data were statistically analyzed with GraphPad Prism software employing a one-way Analysis of variance (ANOVA) method and the Bonferroni multiple comparison test; *p* <0.05 was considered statistically significant.

### cDNA Probes and *In Situ* Hybridization

For *in situ* hybridization, the following complementary DNA (cDNA) probes were utilized: mouse *Bmp7*, and *Ihh*.

Digoxigenin-11-Uridine-5'-triphosphate (UTP)-labeled single-stranded RNA probes were prepared using a DIG RNA labeling kit (Roche Applied Science) according to the manufacturer’s instructions. Tissue sections on slides were treated with 1 μg/mL proteinase K (preincubated at 37°C) for 5 min at room temperature, then washed with PBT, and incubated with hybridization buffer at 65°C for 15 min, after which the corresponding Digoxigenin-labeled probe was added and incubated overnight at 65°C. The following morning, the sections were washed and incubated with FITC-labeled anti-digoxigenin antibodies at 4°C overnight. The following day, the sections were washed and observed under a Zeiss Fluorescence Axio Imager A-1 Microscope and were photographed with an AxioVision Hrc camera (Zeiss, Germany).

## Results

### ECM Alterations and Fibrosis-Related Integrin Expression During Early Stages of OA

**Fig 1:**
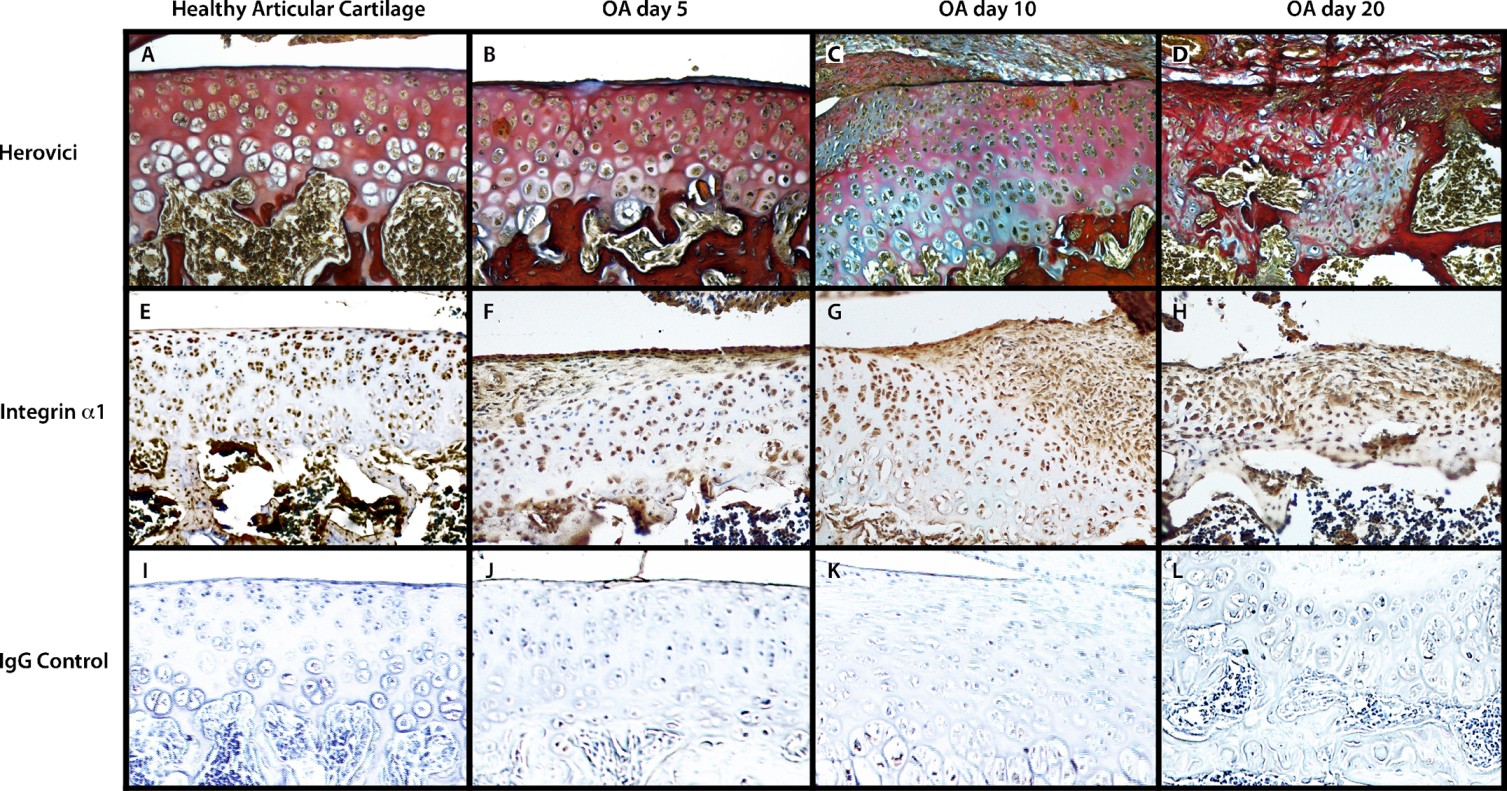
Extracellular matrix (ECM) re-changes during OA. Herovici stain (A–D) showed mature collagen in red and young collagen in blue, immunohistochemical staining for α1 integrin (E–H) and IgG control (I-L); exhibited integrin α1 in the fibrotic area; in healthy articular cartilage (A, E, I); during OA at day 5 (B, F, J), at day 10 (C, G, K), and day 20 (D, H, L).

To evaluate the progression of OA in the rat model, specific staining allows visualizing changes in ECM during disease in histological sections (Fig 1). Safranin-O showed a progressive decrease in proteoglycan content during OA development, indicating the loss of normal phenotype in articular cartilage. In OA, an increase was reported in the expression of Metalloproteases (MMP) such as MMP-13 [32,33], degradation of type IIB collagen [34], and an increase of type I collagen. Fibrous tissue formation can be identified due to accelerated and exacerbated synthesis of type I collagen in early phases of OA, a process known as fibrosis. When employing the Herovici stain, we found that more tightly packed and stable mature collagen fibers stained red fibers and that the least tightly packed or interwoven young collagen fibers were stained in blue. These blue areas have a higher turnover rate, suggesting that such a turnover occurs mainly at the chondrocyte periphery and in the most affected or fibrotic areas of the cartilage (Fig 1A–1D). Consistent with this result, α1β1 integrin was expressed during development of OA in articular chondrocytes in healthy cartilage and in pre- and hypertrophic chondrocytes in late disease stages. In addition, in fibrotic areas, we found a higher expression of α1, α2, and α3 integrins, the typical collagen receptors; we show only α1 integrin pattern expression (Fig 1E–1H). These integrins are evident in healthy cartilage (Fig 1E), but were most evident in the fibrotic area at days 10 and 20 in rats with OA (Fig 1H).

### α5β1 Integrins Were Expressed in Healthy Articular Cartilage and αV Integrin Is Associated with Articular Cartilage Damage in Late OA Stages

**Fig 2:**
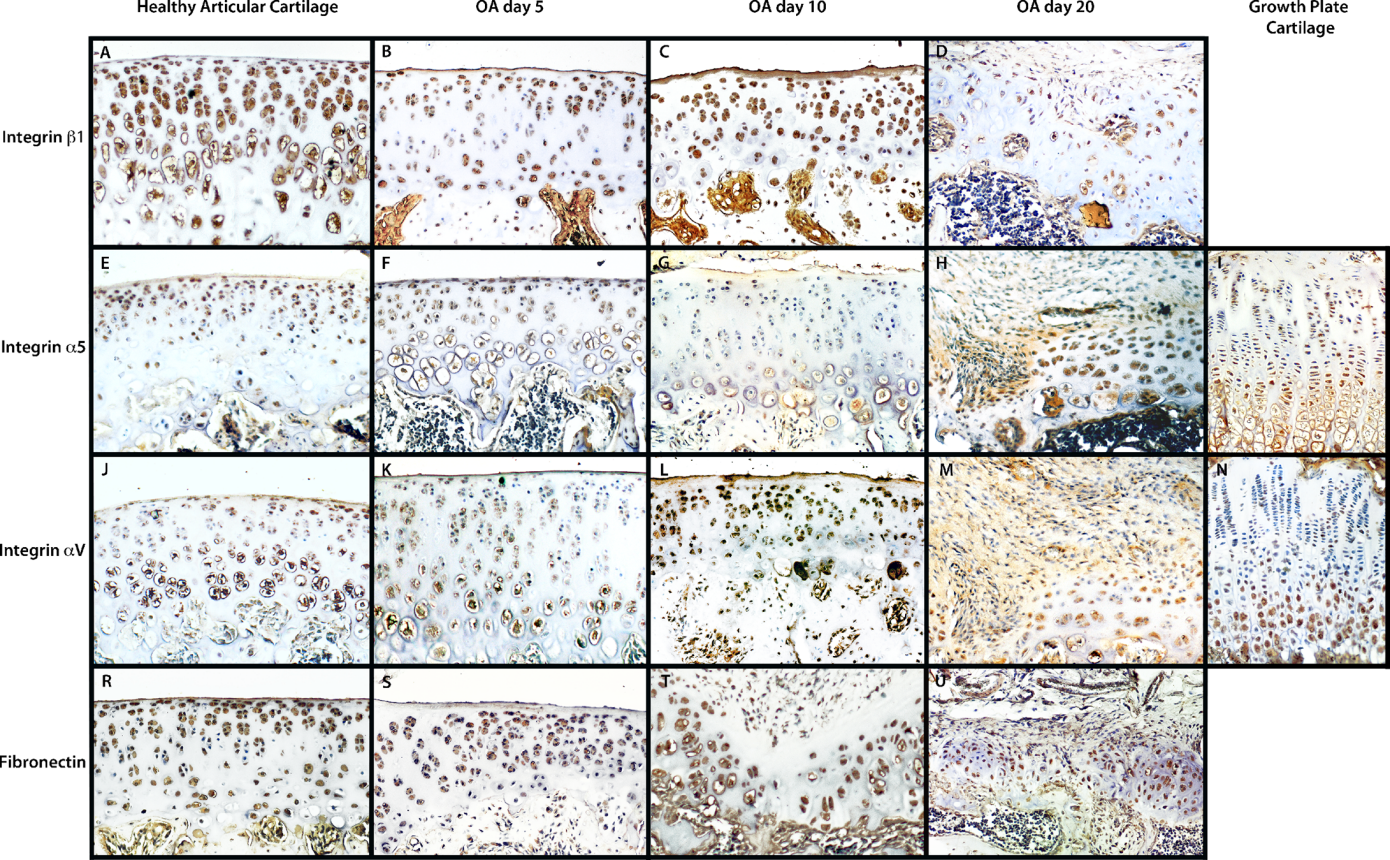
Integrin α5 is expressed in healthy articular cartilage surface, and integrin α V on osteoarthritic articular cartilage recapitulates chondrocyte differentiation, as in the in cartilage growth plate. Immunohistochemical stain for β1 (A–D), α5 (E–H), αV (J–M) integrins; in healthy articular cartilage (A, E, J); during Osteoarthritis (OA) at day 5 (B, F, K), day 10 (C, G, L), and at day 20 (D, H, M), and in the growth plate cartilage, α5 integrin (I) and αV integrin (N). Fibronectin is localizes in the pericellular area of chondrocytes (R-U).

Because the α5 integrin promotes chondrocyte prehyptertrophy during establishment of the growth plate and the αV integrin could be involved in blood vessel recruitment, we evaluated the presence of the sub-units of this integrin during the progress of experimental OA in rat (Fig 2).

Expression of α5 integrin is more evident on the surface of healthy articular cartilage, but was decreased in experimental OA rats from the early stages of OA (Fig 2F); however, in the late OA stages the α5 integrin expression was expressed in the cell cloning zones of cartilage (Fig 2H). Although αV integrin was expressed abundantly in healthy articular cartilage, its expression pattern differs from that of α5 integrin; while α5 integrin is expressed on the surface of articular cartilage (Fig 2E), αV integrin was expressed in the middle or deeper zones (Fig 2J). Similarly, expression of α5 integrins in the growth plate was found in proliferating and prehypertrophic chondrocytes (Fig 2I), while αV integrin was expressed in pre- and hypertrophic chondrocytes (Fig 2N). In OA, αV integrin was expressed strongly on the articular cartilage surface during the middle stage of OA (Fig 2L) and in ossification regions in the late stage of OA (Fig 2M). The α5 integrin was not expressed in fibrous tissue in the late stage of OA (Fig 2H), while αV and β1 integrins were expressed in this healing area in the late stage of OA (Fig 2D and 2M). In addition, β1 integrin expression was abundant in healthy articular cartilage and its expression decreases with the progress of OA (Fig 2A).

Although chondrocyte adhesion to type II collagen comprises a key factor for chondrocyte differentiation, chondrocytes shown a higher-level expression of the typical receptors for fibronectin and vitronectin (α5 and αV integrins) than the typical collagen receptors. Fibronectin protein was found in the pericellular space of chondrocytes in articular cartilage (Fig 2R) and in prehypertrophic chondrocytes (Fig 2T), suggesting that the adhesion of chondrocytes to type II collagen may occur through an arginine-glycine-aspartic acid (RGD) peptide or through fibronectin.

### GDF5 and BMP7 Pattern Expression

**Fig 3:**
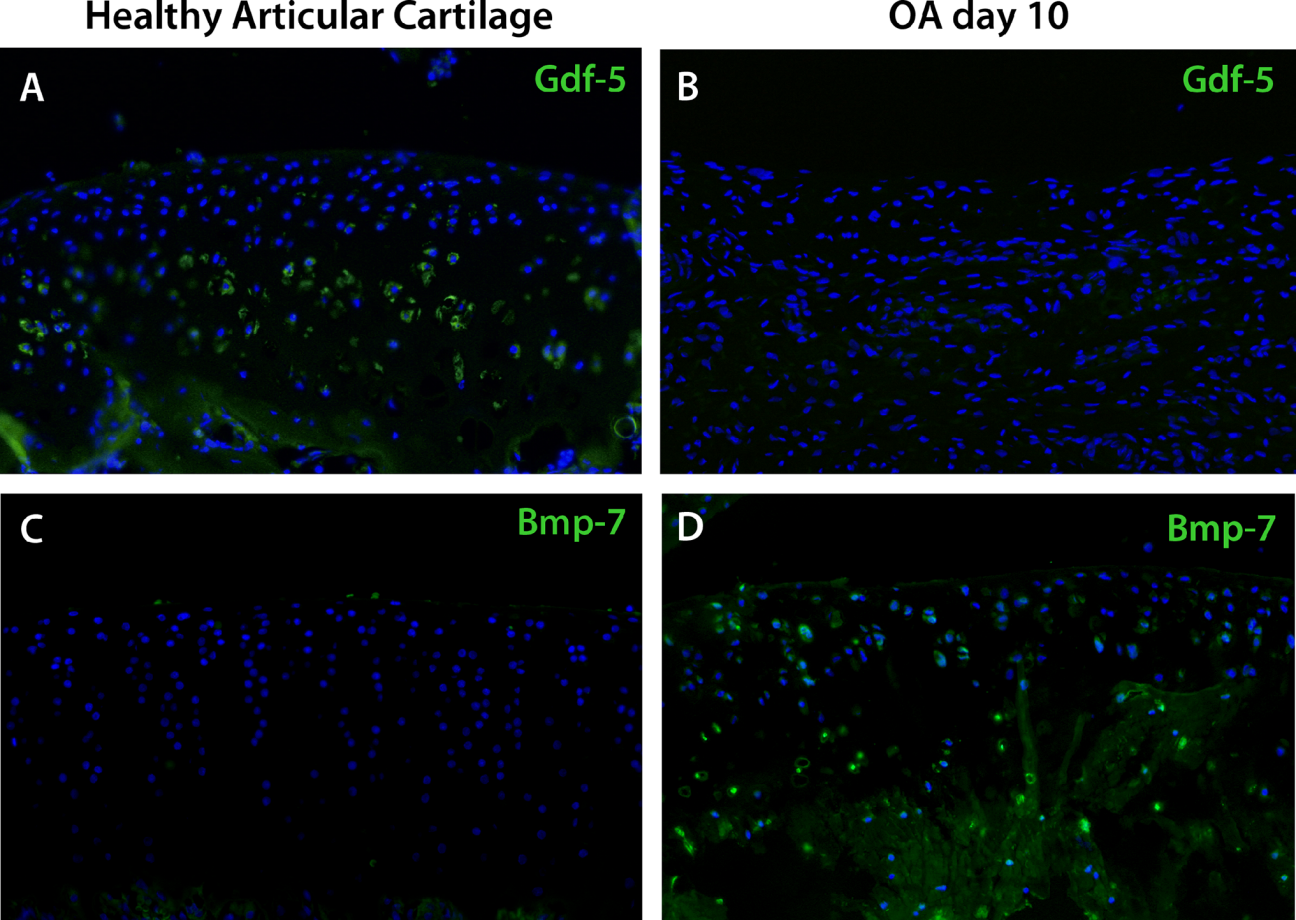
Gdf-5 is associated with healthy articular cartilage and Bmp-7 is associated with OA. Immunofluorescence for Gdf-5 (A, B) and *in situ* hybridization for *Bmp-7* (C, D) in healthy rat articular cartilage (A, C) and at day 10 of OA (B, D) showed Gdf-5 expression in prehypertrophic chondrocytes in healthy articular cartilage. *Bmp-7* is associated with OA from day 10 post-surgery.

BMP-7 and GDF-5 are members of the BMP family whose growth factors control chondrocyte differentiation at multiple levels. We evaluated the expression of both molecules during the development of experimental OA (Fig 3). In healthy articular cartilage, GDF-5 expression was evident in prehypertrophic cartilage (Fig 3A); in contrast, *Bmp-7* expression was absent (Fig 3C). On day 10 of OA, GDF-5 expression was not evident (Fig 3B) and *Bmp-7* was expressed in the damaged articular cartilage region in OA (Fig 3D). We suggest that GDF-5 is related with healthy articular cartilage, while Bmp-7 is related with ossification and cartilage damage during OA.

### Gdf-5 Induces α5β1 Integrin Expression and BMP-7 Induces the αV Integrin

**Fig 4:**
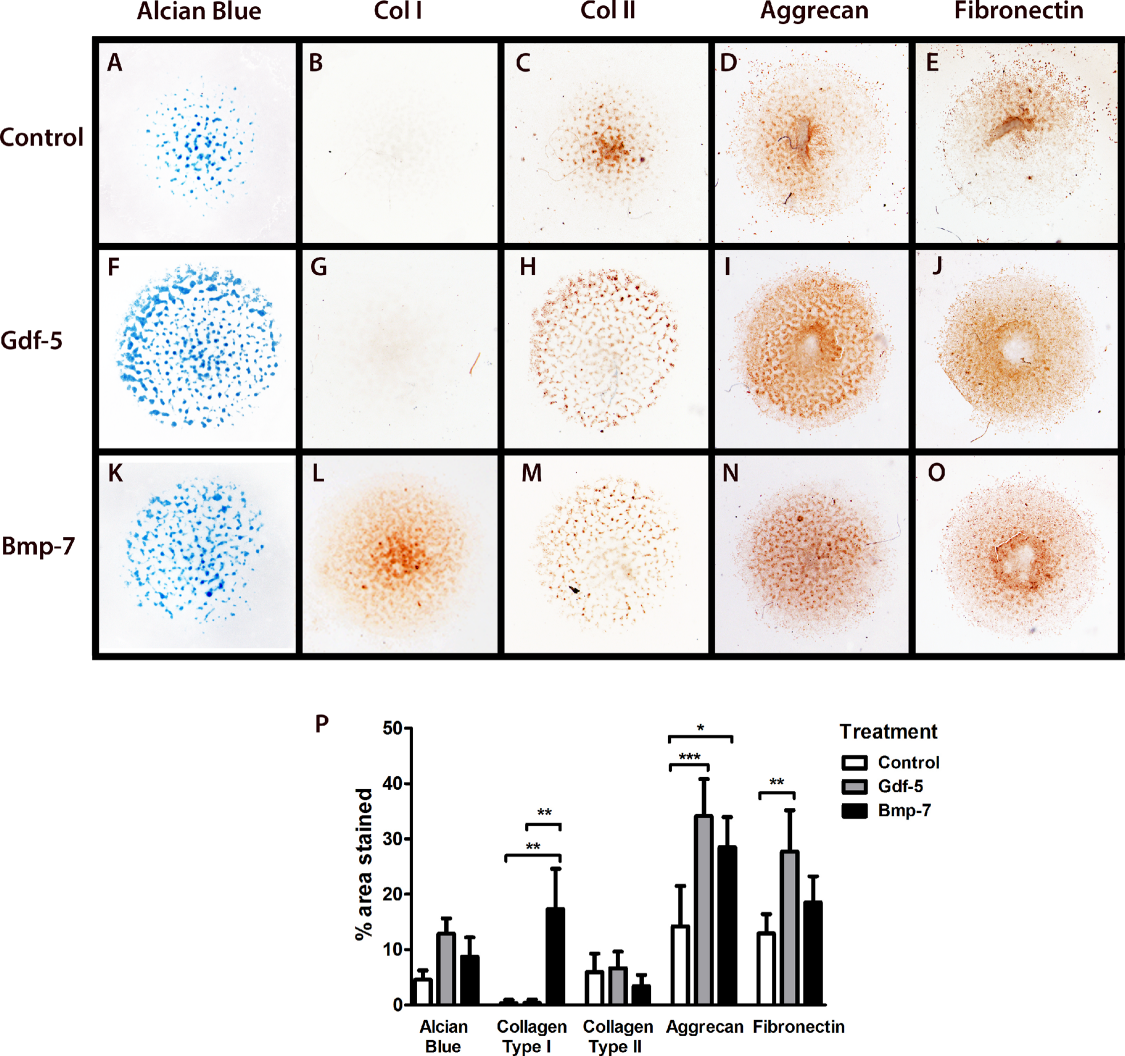
Regulation of Extracellular matrix (ECM) composition by Bone morphogenetic protein (BMP) family growth factors during chondrogenesis. Mouse micromass cultures without treatment (A–E), Gdf-5 treatment (F–J), and Bmp-7 treatment (K–O); Alcian blue stain (A–K) showed cartilage nodules, Immunohistochemical stain for type 1 collagen (A, F, K), type II collagen (C, H, M), aggrecan (D, I, N), and fibronectin (E, J, O). Statistical analysis for histochemical and immunohistochemical data, (P) = percentage of positive area for Alcian blue, type I collagen, type II collagen, aggrecan, and fibronectin stains in micromasses with different treatments; mean values are shown with Standard deviations (SD) (*n* = three independent experiments), One-way Analysis of variance (ANOVA). ***P ˂0.0001; **p ˂0.001; *p ˂0.05). Gdf-5 mainly induces aggrecan expression, type II collagen, and fibronectin; aggrecan is an articular cartilage marker; in contrast, Bmp-7 induce type I collagen, an extracellular component of hypertrophic cartilage and bone.

**Fig 5:**
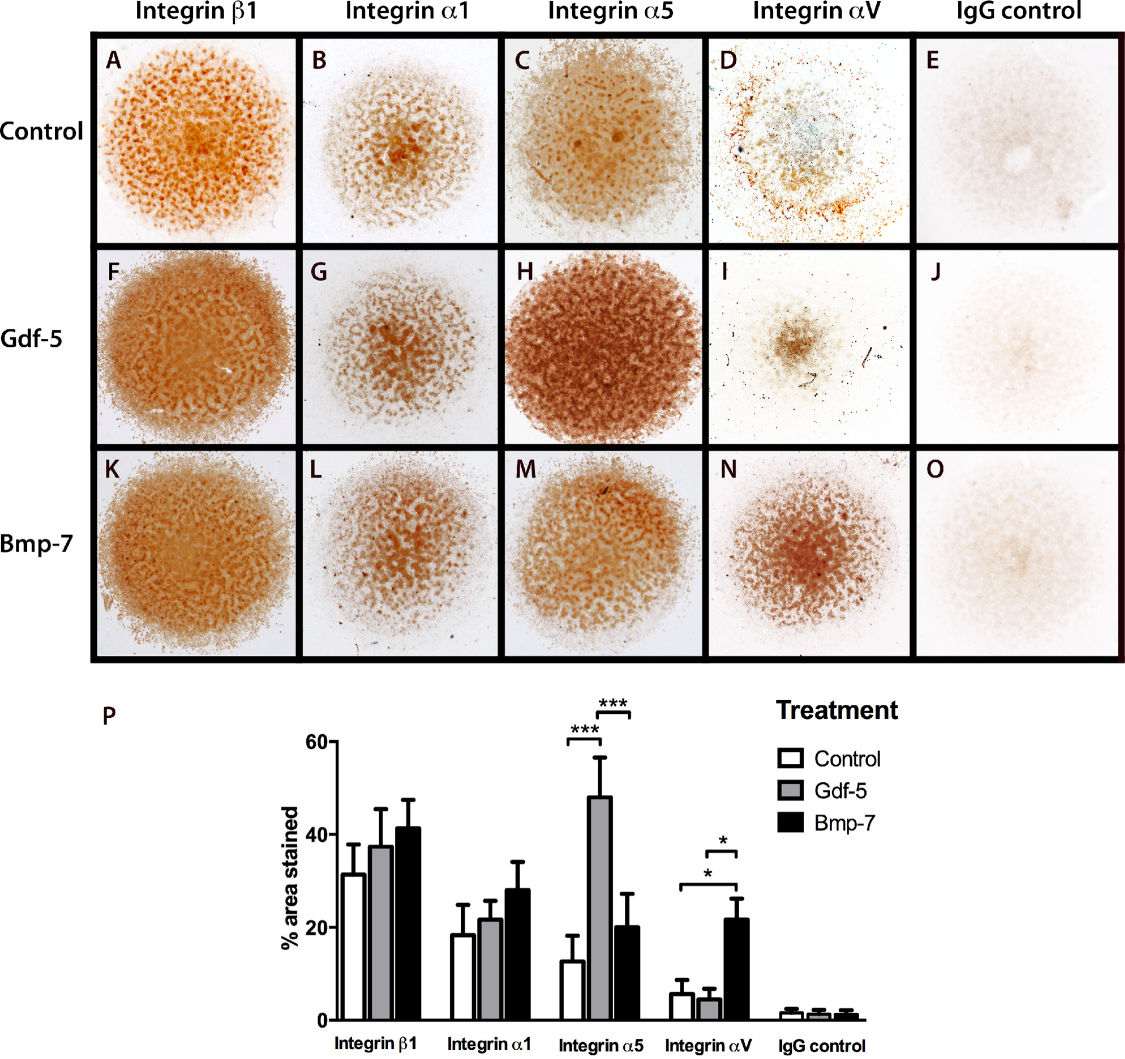
Regulation of integrin expression by Growth differentiation factor (Gdf)-5 and Bone morphogenetic protein (Bmp)-7 during chondrogenesis. Mouse micromass cultures without treatment (A–E), Gdf-5 treatment (F–J), and Bmp-7 treatment (K–O). Immunohistochemical stain for β1 (A, F, K), α1 (B, G, L), α5 (C, H, M), and αV (D, I, N) integrins, IgG control for Immunohostochemestry (E, J, O). Statistical analysis for integrin immunohistochemistry data (P), percentage of positive area for integrin β1, integrin α1, integrin α5, integrin αV and IgG control stains in micromasses with different treatments (control, GDF-5, and BMP-7); mean values are shown with Standard deviations (SD) (*n* = three independent experiments). One-way Analysis of variance (ANOVA). ***P ˂0.0001, **p ˂0.001, *p ˂0.05. Micromass cultures with Gdf-5 treatment induce α5 integrin expression and Bmp-7 treatment induces αV integrin expression.

When considering the mechanism that allows the cartilage to maintain its phenotype and delay hypertrophy, we tested the effect of two growth factors from the family of TGF-β family (Gdf-5 and Bmp-7) on micromass cultures (Figs 4 and 5). Treatment of micromasses with Gdf-5 induced stronger expression of aggrecan (Fig 4I), a molecule characteristic of articular cartilage, while Bmp-7 induced type I collagen and fibronectin expression (Fig 4L and 4O). Treatment with GDF-5 mainly induced stronger α5 integrin expression (Fig 5H), and Bmp-7 induced expression of the αV integrin (Fig 5N). The αV integrin has been related with vasculogenesis in rheumatic diseases [35] and could be implicated in the maturation of the chondrocyte toward hypertrophy. Based on these results, Gdf-5 might be related with articular cartilage maintenance by inducing aggrecan (the main ECM in articular cartilage) and α5 integrin expression. This integrin is related with chondrocyte differentiation during skeletal development and regulates joint formation.

### α5β1 Integrin Are Related with Chondrocyte Maturation in the Articular Cartilage

**Fig 6:**
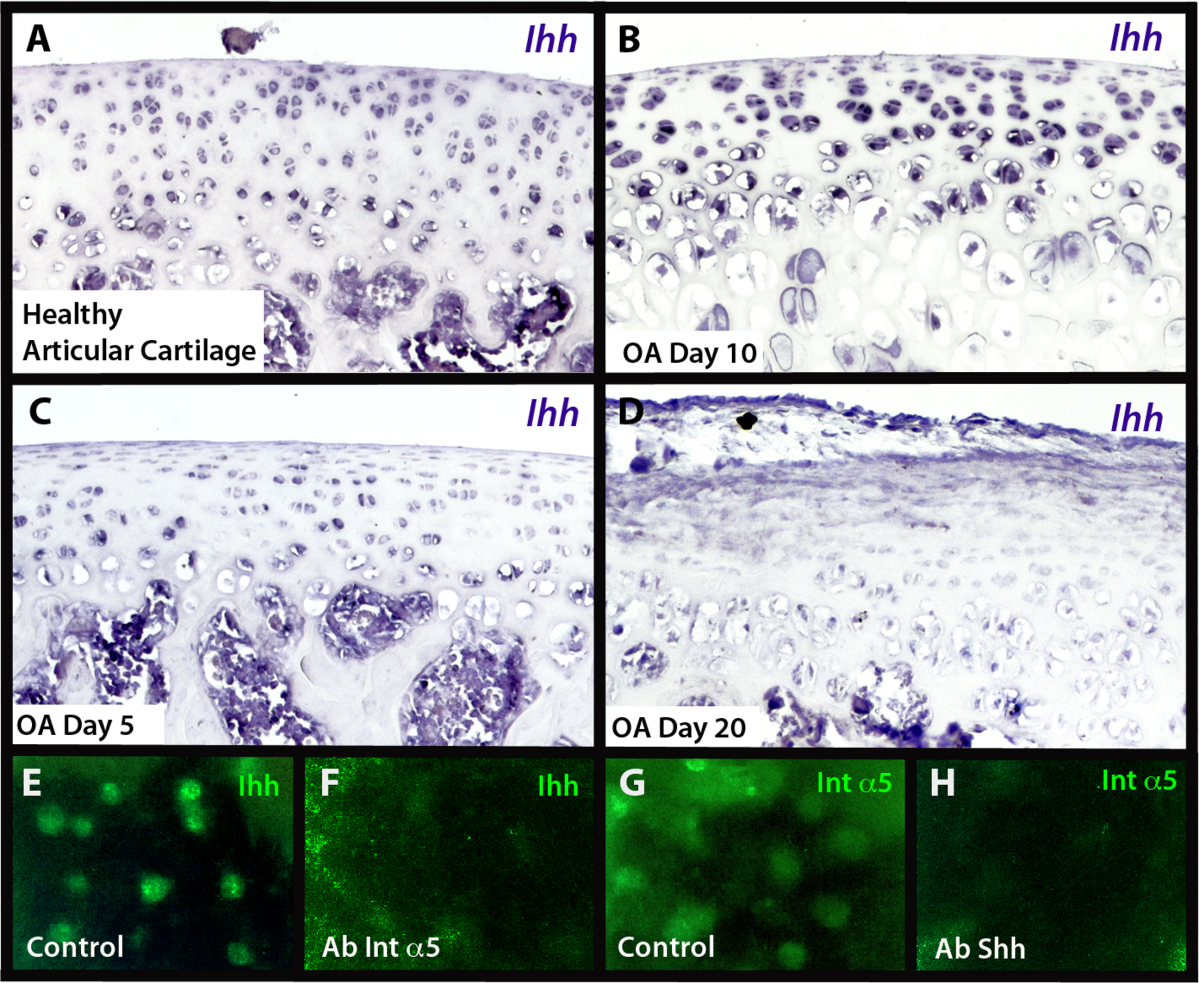
Regulation between α5 integrin and Ihh signaling. *In situ* hybridization for Indian hedgehog (*Ihh*) gene (A–D), in healthy articular cartilage (A), during OA at day 5 (B), at day 10 (C), and at day 20 (D) showed decrease of *Ihh* gene expression in Osteoarthritis (OA). Immunofluorescence in mouse micromass cultures (E–H), without treatment (E, G) showed expression for Ihh (E) and integrin α5 (F); treatment micromasses with blocking antibodies (G, H) for integrin α5 inhibited Ihh expression (F), and treatment with blocking antibodies for Ihh inhibits integrin α5 expression (H).

α5 Integrin expression co-localizes with *Ihh* expression during the growth of long bones and in growth plate cartilage. *Ihh* is a key molecule for the regulation of chondrocyte hypertrophy. Ihh induces chondrocyte proliferation, and its downregulation stops chondrocyte proliferation and subsequently induces hypertrophy maturation. Overexpression of α5 integrin inhibits joint formation during skeletogenesis and induces *Ihh* expression [16]; this mechanism is the basis of chondrocytes maturation to hypertrophy and controls joint formation. Hypertrophy is inhibited at healthy articular cartilage, while articular chondrocytes express α5 integrin and *Ihh* (Figs 2E and 6A). *Ihh* expression was initially enhanced during OA (Fig 5B), and then, Ihh expression is inhibited at late stages of OA (Fig 6C and 6D). This is because Ihh plays an important role in chondrocyte maturation in articular cartilage, regulating the hypertrophy rate in articular chondrocytes. Also, micromass treatment with blockage of anti-α5 integrin 1 μL/mL inhibits Ihh expression (Fig 6F), and micromass treatment with anti-shh inhibits integrin α5 expression (Fig 6H), suggesting a co-regulation between α5 integrin and Ihh signaling.

## Discussion

In this study, we determined the expression pattern of different integrin sub-units of the β1 family and correlated the importance of α5 and αV integrins during articular chondrocyte maturation and the development of OA. It is known that both the α5 integrin and Ihh control the proliferation and prehypertrophy of chondrocyte differentiation [5–13,24,36–38]. The mechanism of these signals appears to be different in articular cartilage as compared with the growth plate (hypertrophic). In previous studies, it was shown that α5 integrin is necessary for prehypertrophic chondrocyte differentiation toward hypertrophy during skeletal growth [14,39]. However, we found that α5 integrin was expressed abundantly on the surface of healthy articular cartilage (Fig 2E) and that it gradually decreased in OA cartilage (Fig 2F, 2G and 2H). This suggests that α5 integrin might play a relevant role in the maintenance of normal articular cartilage, but this is apparently contradictory, because it was reported that α5 integrin is necessary for the differentiation of prehypertrophic chondrocytes into the hypertrophic phenotype, although articular cartilage does not undergo ossification in healthy individuals. We think that there could be a mechanism that controls hypertrophy in articular chondrocytes associated with α5 integrin expression, and that this mechanism is lost at chondrocyte niches during intermediate OA stages (20 days); these cells express α5 integrin again (Fig 2H) and continue differentiating to finally form bone tissue and give rise to osteophytes at a late OA stage. Furthermore, integrin αV was expressed in articular cartilages with OA at regions with damage, and eventually these areas formed bone tissue with blood vessels. Previously, integrin αV was shown to be involved in vasculogenesis in several tumor types [40,41], suggesting a role of integrin αV in chondrocyte hypertrophy and vasculogenesis in which OA occurs with endochondral ossification. Previously, hypertrophy inhibition was observed by overexpression of integrin α5 [16], and a similar effect was shown with *Ihh* overexpression in skeletal development [15,42]. This means that integrin α5 is necessary for chondrogenesis, chondrocyte proliferation, and differentiation, but that its expression should be downregulated to chondrocyte hypertrophy and similarly with Ihh signaling. In contrast, integrin αV may be necessary in chondrocyte hypertrophy during cartilage and bone development and during OA, recapitulating endochondral ossification. Integrin αV could also play a role related with blood vessel invasion into these ossification regions, as proven in metastasis [16,43].

We suggest that co-expression of Ihh and integrins is fundamental for cartilage maintenance in the presence of Gdf-5, because it maintains the articular cartilage phenotype, as proven by the abundant expression of aggrecan, type II collagen, Ihh, and α5 integrin in Gdf-5-cultured micromass. Conversely, when chondrocytes co-express α5 integrin and Ihh in an environment favoring their interaction with Bmp-7, chondrocytes mature into hypertrophy and eventually lose the expression of integrin α5 and Ihh, while Bmp-7 induces integrin αV expression. The mechanism in the switch in the expression of type II collagen and aggrecan for type I collagen, type X, in endochondral ossification could be similar in OA. In endochondral ossification and OA, interactions between chondrocytes and the ECM by means of αV integrin are important and might be related with vasculogenesis, necessary for osteoblast cell migration. This opens an interesting horizon in the field of modified cell lineages that might be applied to the design of tissues for articular cartilage repair.

Finally, regulation of Ihh expression by GDF-5 and integrin α5 is relevant in articular cartilage physiology. Any distortion in these triads signaling on articular cartilage leads to the loss of *Ihh* expression and the articular chondrocyte phenotype, precisely as occurs in OA.
